# Molecular mechanism of Cu metal and drought stress resistance triggered by *Porostereum spadiceum* AGH786 in *Solanum lycopersicum* L.

**DOI:** 10.3389/fpls.2022.1029836

**Published:** 2022-11-10

**Authors:** Falak Naz, Muhammad Hamayun, Mamoona Rauf, Muhammad Arif, Sumera Afzal Khan, Jalal Ud-Din, Humaira Gul, Anwar Hussain, Amjad Iqbal, Ho-Youn Kim, In-Jung Lee

**Affiliations:** ^1^ Department of Botany, Abdul Wali Khan University Mardan, Mardan, Pakistan; ^2^ Department of Biotechnology, Abdul Wali Khan University Mardan, Mardan, Pakistan; ^3^ Centre of Biotechnology and Microbiology, University of Peshawar, Peshawar, Pakistan; ^4^ Department of Food Technology, Abdul Wali Khan University, Mardan, Pakistan; ^5^ Smart Farm Research Center, Korea Institute of Science and Technology, Gangneung, South Korea; ^6^ Department of Applied Biosciences, Kyungpook National University, Daegu, South Korea

**Keywords:** Cu toxicity, heavy metal stress, metallothionine, drought stress, bioremediation, endophytic fungi

## Abstract

Rapid industrialization and global warming have threatened the plants with multiple abiotic stresses, such as heavy metals and drought stress. For crop cultivation, the conventional approach of cleaning the soils by excavation is very costly and not feasible for large scale. Establishing toxin-free and drought-resistant crops is a major challenge in the environment under natural and anthropogenic pressure. In the past decades, copper contamination of agricultural land has become an emerging concern. For dry land reclamation, several new strategies, including bioremediation (phytoremediation and microbial remediation), have been used. Owing to the potential of Cu hyperaccumulators, the current project aims to enhance the drought tolerance and the phytoremediation potential of *Solanum lycopersicum* L. with the inoculation of copper and 12% polyethylene glycol (PEG)–induced drought stress–tolerant endophytic fungus *Porostereum spadiceum* AGH786 under the combined stress of copper heavy metal and PEG-induced drought stress. When *S. lycopersicum* L. was watered with individual stress of copper (Cu) concentration (400 ppm) in the form of copper sulfate (CuSO_4_.5H_2_O), 12% PEG–induced drought stress and the combined stress of both negatively affected the growth attributes, hormonal, metabolic, and antioxidant potential, compared with control. However, the multistress-resistant AGH786 endophytic fungus ameliorated the multistress tolerance response in *S. lycopersicum* L. by positively affecting the growth attributes, hormonal, metabolic, and antioxidant potential, and by restricting the root-to-shoot translocation of Cu and inducing its sequestration in the root tissues of affected plants. AGH786-associated plants exhibited a reduction in the severity of copper (Cu) and drought stress, with higher levels of *SlCOPT (Cu transporters)* and *SlMT (metallothionine)* gene expressions in root and shoot tissues, indicating that AGH786 contributed to resistance to copper metal toxicity and drought stress in the host *S. lycopersicum *L.

## Highlights

- The *P. spadiceum* AGH786 endophytic fungus has been identified as a heavy metal stress–resistant, Cu hyperaccumulator, and drought stress–tolerant fungus in previous research.
- The *P. spadiceum* AGH786 endophytic fungus promoted growth and alleviated the combined stress of Cu and drought in *S. lycopersicum* L.
- The *P. spadiceum* AGH786 association enhanced the level of growth-promoting hormones, metabolites, and antioxidants under the combined stress of Cu and drought in *S. lycopersicum* L.
- The gene expressions of *SlCOPT (Cu transporters)* and *SlMT (metallothionine)* were strongly induced by *P. spadiceum* AGH786 inoculation in *S. lycopersicum* L. plants under the combined stress of Cu and drought.
- With the induction of *SlCOPT (Cu transporters)*, *P. spadiceum* AGH786 restricted the uptake and translocation of Cu from root to shoot tissues and sequestered the toxic Cu ions in fungal biomass and root tissues of *S. lycopersicum* L.
- The AGH786*–S. lycopersicum* association proved to be an effective combination of myco- and phytoremediation strategies for quickly reclaiming heavy metal–contaminated soils in drought-prone areas.

## 1 Introduction

Heavy metal contamination is increasingly becoming an environmental problem and causes great adverse effects around the world in the form of inorganic pollutants, which are discarded in our soil and water and into the atmosphere because of increased population growth and demands on rapidly growing metal industries, agriculture, fertilizers, pesticides, and improper waste disposal (Briffa et al., 2020). The exposure of plants to soil contamination by metal stress aggravates drought stress in an additive manner, making the plants more vulnerable to drought. Moreover, drought and heavy metal stress undesirably disturb soil fertility too, which retards the growth and development of plants (de Silva et al., 2012).

Copper (Cu) is an essential metal for normal plant development but becomes rapidly toxic in excess. For example, when the soil materials have been rich in copper and the pH of the soil offers metal availability if the soil has been contaminated by coal mining and waste deposits, or when agricultural soils have been heavily fertilized with manure or sewage, a high Cu content in the soil, which is toxic, may occur (Rehman et al., 2020; Srivastava et al., 2021). Cu is an essential micronutrient required for plant growth and is a good component of enzymatic activity, protein synthesis, and several biochemical processes in the cell. For example, it is the cofactor of enzymes involved in many biochemical processes, including photosynthesis, respiration, detoxification of peroxide anions, ethylene perception, and cell wall metabolism. The natural soil’s Cu content ranges from 60 to 125 mg kg^−1^ (Kabata-Pendias, 2010).

The average content of Cu in plant tissues ranges from 2 to 50 μg g^−1^ dry weight (Cohu and Pilon, 2007). Cu is highly toxic as the redox cycling between Cu(I) and Cu(II) catalyzes the production of hydroxyl radicals *via* Fenton’s reaction (Dra̧zkiewicz et al., 2004). Symptoms of toxicity usually appear when the Cu concentration exceeds 20 μg g^−1^ dry weight in vegetative tissues (Marschner, 1995). The more typical symptoms of copper toxicity are leaf chlorosis and reduced growth, which are mainly caused by nutrient uptake inhibition or actual contact with plant metabolism (Kumar et al., 2021; Angulo-Bejarano, et al., 2021). Cu is a heavy metal anthropogenic contaminant that causes major health problems and affects plants; its toxic level affects their growth and productivity. Plant reaction to metal-induced stress may involve the synthesis of various secondary metabolites (Chrysargyris et al., 2021). Crop cultivation on such contaminated types of soil affects plant growth and productivity by damaging photosynthesis and inhibiting transpiration and water uptake.

Plants have been known to adopt different strategies under multiple stressful growth environments to enhance their tolerating potential by evolving various physiological, morphological, biochemical, and molecular mechanisms. Physiologically, plants can reorganize their root system architecture by inducing primary root growth inhibition and an increase in the lateral root density. Although the morphological changes are generic, they may not be induced through the same signaling pathway. Plant hormones, mainly auxin, cytokinin, and ethylene, control root system architecture and remodel characteristics of the root, including primary root and lateral root growth as well as root hair formation (Juraniec et al., 2016). Moreover, another adaptive mechanism is root colonization, which is a competitive process and a vital step in the creation of plant–microbe relationships, and both host plants and their associated microbes’ characteristics may affect it (Reinhold-Hurek and Hurek, 2011).

For dry, contaminated land reclamation, several new strategies, including bioremediation (phytoremediation and microbial remediation), have been used. Phytoremediation emerged as a promising cost-effective and environmentally friendly technology to render metals less bioavailable and less toxic (phytostabilization); clean up metal-polluted soils (phytoextraction); and/or uptake and release metals in methylated, volatile forms to the atmosphere such as mercury, selenium, and arsenic (phytovolatilization). The most employed strategies are phytoextraction and phytostabilization (de Silva et al., 2012; Nascimento et al., 2021). However, for phytoextraction (natural and chemical-assisted phytoextraction), several hyperaccumulator crops uptake and overaccumulate the heavy metal in their edible parts and medicinally used plant tissues, which is a major limitation and a serious health concern for human and animals. However, researchers have also used microbial remediation of the contaminated soils.

More recently, phytoextraction with aided microbial remediation has proven as more effective strategy for the remediation of heavy metals from the environment, as microbes not only self-accumulate metals but also help host plants in metal accumulation in root tissues by restricting the uptake and translocation by binding them to extracellular and intracellular molecules. For example, plant growth-promoting bacteria *Kluyvera intermedia*, *Klebsiella oxytoca*, and *Citrobacter murliniae* isolated from a site contaminated by gold ore processing activities to assist the phytoremediation of As, Cd, and Pb by *Sorghum bicolor* and mitigate the metal toxicity in plants. (Boechat et al., 2020). In recent years, the absorption of copper and other heavy metals through filamentous fungi has received a great momentum as an evolving technology for the elimination of the heavy metals from mining and industrial waste (Dhankhar and Hooda, 2011; Ahemad and Kibret 2014), for example, Cu heavy metal–tolerant *Rhizopus microspores* (Oladipo et al., 2018), *Aspergillus niger* and *Penicillium citrinum* (Sazanova et al., 2015), *Postia placenta, Meruliporia incrassate, Wolfiporia cocos*, and *Antrodia vaillantii* (Clausen and Green, 2003), *Laccaria bicolor* (Reddy et al., 2014), and Cd heavy metal–tolerant *Cerrena unicolor* (Jarosz-Wilkołazka, 2006).

The heavy metal toxicity inhibits enzymatic activity, plant growth, and yield (Nematshahi et al., 2012). To withstand heavy metal stress and metal toxicity, plants also have evolved various defense mechanisms, such as (1) reduced heavy metal uptake; (2) metal sequestration in vacuoles, both extracellular and intracellular; (3) detoxification by enzymes; (4) regulating excessive metal ion homeostasis; (5) binding to phytochelatins/metallothioneins (MTs); (6) activation of various antioxidants, enabling them to survive in the presence of a high concentration of copper; (7) upregulation of copper-induced genes through Cu signaling; and (8) overaccumulation of Cu-resistant proteins (Juraniec et al., 2016; Kramer et al., 2020).

Napoli et al. (2019) found that *Solanum lycopersicum* L. appears to be one of the efficient phytoremediator plants in the removal of Cu concentration from the soil, considering the total uptake by the plant and the remarkably accumulated Cu in fruits and roots. However, being an edible food crop, an alternative strategy must be used for the cultivation of *S. lycopersicum* L. in heavy metal–contaminated, multistress-prone regions. So that soil can be eliminated side by side, contamination-free crops must be produced by farmers.

Combining plants and their associated microorganisms to eliminate contaminants has proven to be a cost-effective, *in situ*, and promising technology (Tiodar et al., 2021), as genetically and physiologically resistant endophytic fungal microbes have shown the dominant potential to increase the remediation of heavy metals and stress tolerance in plants (Aziz et al., 2021a; Aziz et al., 2021b). Given the serious challenges posed by global industrialization to crop cultivation, as well as the risk of phytoremediation by major edible crops such as tomatoes, in a multistress environment, the current study was initiated to investigate a novel strategy for mitigating the harmful effects of combined heavy metal (Cu) and drought stress. Therefore, the present research also deciphers the exploitation of the plant–microbe interaction for multistress alleviation to grow a contamination-free, healthy crop of *S. lycopersicum* L. *under* drought stress.

Endophytes can help the host plant species withstand multiple difficulties, such as heavy metals, drought, high temperature, and salinity, in addition to inducing stress-responsive genes (Rauf et al., 2021; Javed et al., 2022; Ali et al., 2022a; Aziz et al., 2022a; Aziz et al., 2022b; Rauf et al., 2022). Endophytes have sparked a lot of interest in recent years because of their function in host seedlings and defense.

Amin and Ahmad (2015) found that the *S. lycopersicum* L. crop is susceptible to multiple impositions of stresses, such as heavy metal, as well as drought stress. However, research has not been done so far for mitigation of such combined stresses in *S. lycopersicum* L.

Here, we aimed to exploit the combination of microbial extraction, along with phytoextraction, by taking advantage of the endophytic fungus (*Porostereum spadiceum* AGH786) and the host plant *S. lycopersicum* L. as hyperaccumulator of Cu metal. Hence, the current study was rationalized to unravel the multistress-tolerant endophytic fungi *P. spadiceum* AGH786 for the alleviation of the combined stress of Cu and drought in *S. lycopersicum* L. Hence, the present research aimed to explore the effect of *P. spadiceum* AGH786 on the physiological, morphological, hormonal, biochemical, and molecular parameters of *S. lycopersicum* L. grown under the combined stress of Cu metal toxicity and drought. The current investigation enabled us to unravel the dual potential of the *P. spadiceum* AGH786–*S. lycopersicum* symbiotic association as mycoremediation, as well as the phytoremediation of Cu toxicity in dry, contaminated lands, with the growth promotion of the host plant.

## 2 Methodology

### 2.1 Requisition of *P. spadiceum* AGH786

*P. spadiceum* (AGH786, Accession No 786) (Hamayun et al., 2017) was obtained in the form of slants from the Department of Botany, Plant–Microbe Interactions (PMI) Lab, Abdul Wali Khan University Mardan.

#### 2.1.1 Assessment of growth and tolerance response of the *P. spadiceum* AGH786 strain under the stress of Cu metal and polyethylene glycol–mediated drought

Fungal strain *P. spadiceum* AGH786 was refreshed according to the method of Hamayun et al. (2009) and Khan et al. (2009). For subculturing, a section of the fungal colony was transferred on media containing copper (II) sulfate (CuSO_4_.5H_2_O) salt (CAS No. 7758-99-8; Sigma-Aldrich, Deisenhofen, Germany); supplemented with various concentrations of 0, 100, 500, and 1,000 ppm; incubated at 25°C; and kept overnight at 28°C in the dark; growth was evaluated phenotypically compared with control (0 ppm), as described by Shen et al. (2013). The *P. spadiceum* AGH786 strain was grown in a liquid medium by the method (Hamayun et al., 2017) supplemented with various concentrations of 0, 100, 500, and 1,000 ppm. After incubation, the filtrate was used for the analysis of metabolites and metal concentrations, subsequently. The drought tolerance response was also evaluated on PDA plates supplemented with 12% polyethylene glycol (PEG 8000), as described by (Praveen Kumar et al., 2014).

### 2.2 Response of *P. spadiceum* AGH786 to 12% polyethylene glycol–induced drought stress

The drought tolerance response was evaluated as described by (Kumar et al., 2014) using 12% polyethylene glycol (PEG 8000) amended on PDA plates.

### 2.3 Determination of hormones and metabolites in the fungal cultural filtrate

Primary and secondary metabolites (carbohydrates, proteins, and lipids), as well as indole-3-acetic acid (IAA), gibberellic acid (GA), salicylic acid (SA), and abscisic acid (ABA), were determined in the cultural filtrate of *P. spadiceum* AGH786. IAA was estimated by using the Salkowski reagent as described earlier (Hussain and Hasnain, 2011). GA and ABA were determined by the method of Ergün (2002). SA was estimated by using the technique of Warrier et al. (2013). For the determination of the total flavonoid content, the method of El Far and Taie (2009) was used. The method of Malik and Singh (1980) was adapted for the determination of the total phenolic content in fungal culture filtrate. The proline content was determined according to the method of Bates et al. (1973). Total soluble sugars were estimated as described by Nayer and Reza (2008).

### 2.4 Soil experiment

#### 2.4.1 Preparation of soil for the inculcation

Soil (sandy loam) suitable for local cultivation of tomato crops was collected from the Mardan district of Khyber Pakhtunkhwa, Pakistan, for physicochemical analysis. The sand content of the soil mixture ranged from 71% to 74%. Silt content ranged from 11% to 13%. Clay content was 11%–16%. Soil pH ranged from 7.3 to 7.8. The electrical conductivity of the soil mixture ranged from 0.7% to 6%. Organic matter was 1.5%, carbonate was 1.32 meq/L, bicarbonate was 2.8 meq/L, and Cl^−1^ was 15 meq/L.

The sterilized soil was supplemented with fungal mycelium (2 g/100 g of soil), and plastic pots were prepared with 500 g of soil mixture, sufficient enough for growing tomato plants for up to 5 weeks. Pots without fungal biomass were used as control, and the soil pots were kept for 1 week to grow fungal hype uniformly in a growth chamber in the lab at 28°C.

#### 2.4.2 Sowing of *S. lycopersicum* L. seeds

A non-hybrid variety of *S. lycopersicum* L. (Rio Grande) seeds was obtained from the National Agricultural Research Centre, Islamabad. Healthy, mature, and uniform-sized seeds were selected by physical appearance. Seeds were washed three times with autoclaved distilled water. Ethanol (70%) was applied for the sterilization of *S. lycopersicum* L. seeds. The seeds were washed with distilled water thrice and sown in the soil premixed with fungal biomass (2 g/100 g of soil). Then, *S. lycopersicum* L. seed pots were shifted to a growth chamber (day/night cycle: 14 h 28°C ± 0.3°C, 10 h 25°C ± 0.3°C; relative humidity, 70%; six plants per treatment) for 1 month, in November 2019, in the lab at Abdul Wali Khan University Mardan. The experiment was designed with a completely randomized design; there were eight treatments, and each treatment has six replicates.

#### 2.4.3 Experimental design

Treatment 1. Control (distal water)
Treatment 2. AGH786 (2 g/100g) (endophytic fungus)
Treatment 3. PEG (12%) (drought stress)
Treatment 4. Cu (400 ppm)
Treatment 5. Cu (400 ppm) + PEG (12%)
Treatment 6. AGH786 (2 g/100 g) + PEG (12%)
Treatment 7. Cu (400 ppm) + AGH786 (2 g/100 g)
Treatment 8. Cu (400 ppm) + AGH786 (2 g/100 g) + PEG (12%)

Napoli et al. (2019) reported 400 ppm of Cu supplementation for evaluation of Cu uptake and accumulation response of *S. lycopersicum* L. from the Cu-contaminated soil. Consistently, in the current research, *S. lycopersicum* L. plants were selected as an efficient Cu accumulator and supplemented with the copper (II) sulfate (CuSO_4_.5H_2_O) salt (CAS No. 7758-99-8; Sigma-Aldrich, Deisenhofen, Germany), at the concentration of 400 ppm Cu/pot at 14, 21, and 28 days after germination. After 5 weeks, growth parameters of *S. lycopersicum* L. seedlings, including total chlorophyll content, shoot–root length, and fresh and dry weight of shoot–root, were measured. Seedlings were harvested, and fresh leaves grind in liquid nitrogen for total carbohydrates; proteins; lipids; flavonoids; phenols; proline; total antioxidants such as ascorbic oxidase (AAO), catalase (CAT), and peroxidase (POD); and different plant hormones such as GA, IAA, SA, and ABA. Chemicals and reagents were purchased from Sigma-Aldrich (Deisenhofen, Germany), Fluka (Buchs, Switzerland), and Merck (Darmstadt, Germany).

#### 2.4.4 Drought stress on *S. lycopersicum* L.

For induction of drought stress, PEG 8000 was used at 12%, and three doses of 300 ml of the PEG solution (12%) were given to each pot for 3 days (Hamayun et al., 2010).

### 2.5 Growth parameters

At the end of the experiment, the total yield was recorded by measuring the shoot, root length, and fresh and dry weight of *S. lycopersicum* L. seedlings.

#### 2.5.1 Chlorophyll and carotenoids content

For determination of chlorophyll and carotenoid contents, Maclachlan and Zalik’s (1963) method was used.

#### 2.5.2 Determination of endogenous indole-3-acetic acid, gibberellic acid, salicylic acid, and abscisic acid

For the determination of endogenous IAA contents of grained fresh leaves (0.1 g) in liquid nitrogen, purification and extraction of IAA were performed as described above (Hussain and Hasnain, 2011). GA and ABA were determined by the method of Ergün (2002). SA was estimated by using the technique of Warrier et al. (2013).

#### 2.5.3 Determination of secondary metabolites

The total flavonoid content was estimated by the AlCl_3_ method, as mentioned earlier (El Far and Taie, 2009). The phenolic content was determined by the method discussed above. Proline contents were analyzed according to the protocol of Bates et al. (1973). The total soluble sugar estimation was performed according to Nayer and Reza (2008), as discussed above. Optical Density (OD) was noted at 485 nm. For extraction of total lipid, we used the method of Van Handel (1985). The determination of malondialdehyde (MDA) was done as mentioned earlier (Schmedes and Hølmer, 1989).

#### 2.5.4 Determination of antioxidant activities

With minor modifications, the DPPH (1,1-diphenyl-2-picryl hydroxyl) scavenging activity was measured using the method of Abbasi et al. (2011), Plant matter (0.1 g) was mixed in 1 ml methanol, along with a 0.004% methanol solution of DPPH. About 1 ml of the DPPH solution was then added to 0.5 ml of the samples and then blended and kept for 30 min at room temperature in the dark. The intensity of the DPPH staining was estimated to be 517 nm. The decline in absorption by the sample suggested an elevated scavenging of free radicals according to the equation


$$\%DPPH=\left(\frac{1-AE}{AD}\right)\times 100$$

where *AE* = absorption with extract and *AD* = absorption of DPPH solution only.

CAT activity was used for H_2_O_2_ cleavage (Guo et al., 2006). The decrease in H_2_O_2_ is followed by a decrease in absorption at 240 nm, which was measured as M H_2_O_2_ min^−1^ cleavage.

After dehydrogenating guaiacol as a substratum, the generation of PODs was calculated (Malik and Singh, 1980). In 3 ml of the phosphate buffer (pH 7.0), the enzyme was extracted from the plant. First, take 0.1 g of leaves and grind in 1 ml Tris buffer, centrifuged for 15 min at 5°C at 12,000 rpm. Within 2–4 h, the obtained supernatant was employed as an enzyme source. Pipet 3 ml phosphate buffer (0.1 M), 0.03 ml H_2_O_2_ (12.3 mM or 0.04%) solution, 0.05 ml guaiacol solution (20 mM), 0.1 ml plant extract, and 0.03 ml H_2_O_2_ (12.3 mM or 0.04%) solution into a cuvette. The resulting mixture was properly shaken, and OD was recorded at 436 nm.

#### 2.5.5 Reactive oxygen species accumulation through 3,3′-diaminobenzidine

For examining the H_2_O_2_ biosynthesis and the accumulation of the 3,3′-diaminobenzidine (DAB; Sigma, St. Louis, MO, USA), a staining assay was performed using leaf disc, as described by Rauf et al. (2022).

### 2.6 Heavy metal content analysis in fungal biomass and plant tissues

The bioavailability of Cu was assessed using atomic absorption spectrometry, as described earlier (Li et al., 2014). For determining the Cu content in fungus grown on Cu supplemented media, fungal biomass was retrieved by filtering. While plant samples were washed in water to remove surface element traces, then divided into leaf, root, and shoot segments, and oven-dried at 65°C for 48 h until the weight was constant. The samples were then crushed to powder form with a mortar and pestle, then 0.2 g roots/shoots powder was added for digestion with 5 ml HNO_3_ (65% w/w) at 110°C for 2 h, then cooled and mixed with 1 ml H_2_O_2_ (30% w/w), and heated for 1 h. Next, the digests were diluted with deionized water in a conical flask with triple deionized water (Shen et al., 2013).

### 2.7 RNA isolation and cDNA synthesis

Total RNA was extracted from *S. lycopersicum* L. seedlings using the Gene JET Plant RNA Purification Kit (Thermo Scientifics), as specified by the manufacturer. During the isolation procedure, the DNase treatment was carried out using RNase-free DNase that was obtained from the TURBO DNase Kit by Ambion (Cambridge, United Kingdom). Around 2 µg of total RNA was reverse-transcribed using the Revert Aid First Strand cDNA Synthesis Kit by Invitrogen (Karlsruhe, Germany), as described earlier (Rauf et al., 2021).

qPCR primers were designed utilizing Primer 3.0 (Untergasser et al., 2012) for gene expression analysis of the heavy metal stress–related molecular marker genes *copper transporters (SlCOPTs)* and *metallothionein (SlMTs).* As an internal control, *ACTIN2* was used. All primers were synthesized from Bio Basic (Korea), and sequences with gene accession numbers have been mentioned in **Table 1**. Amplification of each gene was performed in triplicate by using an ABI PRISM 7900HT sequence detection system (Applied Biosystems Applera, Darmstadt, Germany), and the amplification product was visualized using SYBR Green (Applied Biosystems Applera, Darmstadt, Germany). Amplification curves were analyzed with a normalized reporter (Rn: the ratio of the fluorescence emission intensity of SYBR Green to the fluorescence signal of the passive reference dye). Reverse transcription–quantitative PCR (RT-qPCR) expression analysis was performed by using three independent biological replicates with at least three technical replicates as described earlier (Rauf et al., 2022).

**Table 1:** Primers used for reverse transcription–quantitative PCR (RT-qPCR).

Primers used for RT-qPCR			
Gene name	Gene accession	Gene code	Primer sequences
*Copper transporter*	*Solyc08g006250*	*SlCOPT1_F*	*ATTCTCTTCTCCGGTTGGCC*
		*SlCOPT1_R*	*CTAACTCCGTACAACGCCGT*
*Copper transporter*	*Solyc06g005820*	*SlCOPT2_F*	*GGCCAACCTGAGAAGAGAATC*
		*SlCOPT2_R*	*ATGAAGAACGACGGCCACAT*
*Copper transporter*	*Solyc09g011700*	*SlCOPT3_F*	*ACAAAAGGCCCATAGGTGCT*
		*SlCOPT3_R*	*TCTCAACCGCGACAAGTTCA*
*Copper transporter*	*Solyc10g084980*	*SlCOPT4_F*	*AAGCCGGAATACAAGCGGTT*
		*SlCOPT4_R*	*CTGCATGACCAACAACAGCC*
*Copper transporter*	*Solyc02g082080*	*SlCOPT5_F*	*GCTGTGAATGCTCCCCTTCT*
		*SlCOPT5_R*	*TGACATCATCCTCATCGCCG*
*Copper transporter*	*Solyc09g014870*	*SlCOPT6_F*	*TGACATGCCAGGAATGGGAG*
		*SlCOPT6_R*	*AGGACATACATGCCCGTTCG*
*Actin*	*Solyc05g054480*	*SlACTIN_F*	*AGATCCTCACCGAGCGTGGTTA*
		*SlACTIN_R*	*GAGCTGGTCTTTGAAGTCTCGA*
*Metallothionein*	*NM_001247117.2*	*SlMT1-F*	*CTAGCTGCAAGTGCGACAAC*
		*SlMT1-R*	*ACCCCAAGCACCAAAGTCTC*
*Metallothionein*	*EU884310*	*SlMT2-F*	*GCTGTGGATCTAGCTGCAAGTGCG*
		*SlMT2-R*	*AAGGGTTGCACTTGCAGTCAGATCC*
*Metallothionein*	*NM_001247125.2*	*SlMT3-F*	*ATGTCTTGCTGTGGTGGAAG*
		*SlMT3-R*	*TAGCAATTGCAAGGGTCACA*
*Metallothionein*	*NM_001247362.2*	*SlMT4-F*	TGTGGGATGTACCCCGACTT
		*SlMT4-R*	TCTGTTGCTTTCTCAGCCACT

### 2.8 Statistical analysis

Each experiment was performed in triplicates, the data were analyzed using ANOVA through SPSS-20, and the means that differed from one another in a significant way were further examined using the Duncan Multiple Range Test at the p-value of 0.05 (SPSS, Inc., Chicago, IL, USA).

## 3 Results

### 3.1 Drought stress and Cu metal toxicity response of *P. spadiceum* AGH786

**Figure 1:**
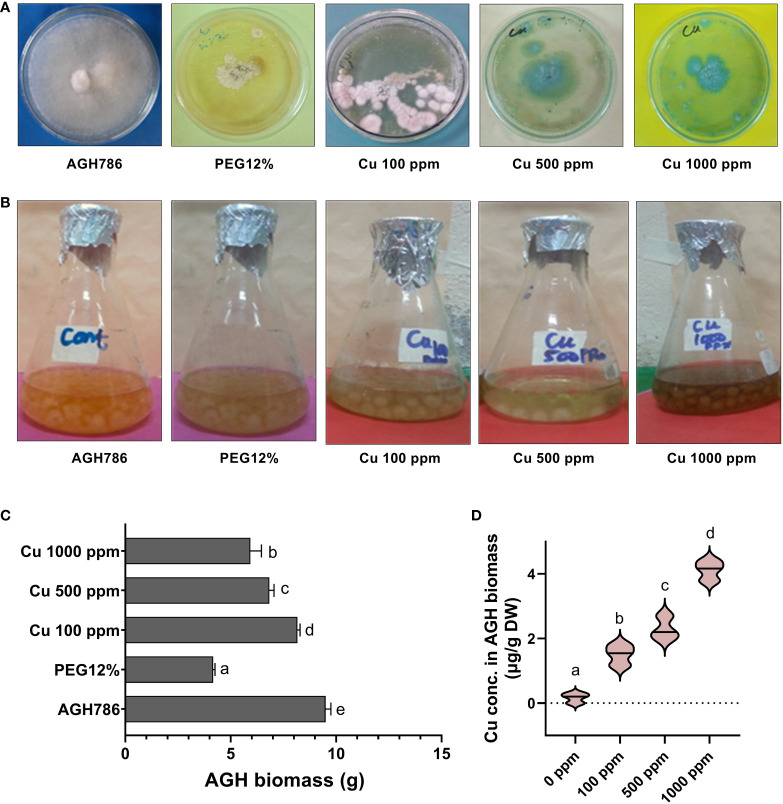
**(A)** Growth of *P. spadiceum* AGH786 on different concentrations of Cu and 12% PEG on solid media. **(B)** Growth of *P. spadiceum* AGH786 on different concentrations of Cu and 12% PEG on liquid media. **(C)** Fresh weight of *P. spadiceum* AGH786 on different concentrations of Cu and 12% PEG on liquid media. **(D)** Bioavailable Cu concentration in fungal biomass. Data represent the mean with standard error, and letters represent the significant difference (p < 0.05).

Tolerance response of *P. spadiceum* AGH786 against drought stress and Cu toxicity has been shown in **Figures 1A, B**, which revealed the differential tolerance potential of the *P. spadiceum* AGH786 strain growing on media (PDA and Czapek), supplemented with the different concentrations of copper salt at 100, 500, and 1,000 ppm and 12% PEG. In the current results, the *P. spadiceum* AGH786 strain showed the highest tolerance potential in terms of sustainable biomass production at 100 ppm copper supplemented media compared with the higher concentrations (500 and 1,000 ppm). In addition, 12% PEG–treated media also showed sufficiently sustainable biomass production (**Figure 1C**). Quantification of bioavailable Cu content revealed that *P. spadiceum* AGH786 mycelium efficiently absorbed the Cu supplemented in growth media, in a dose-dependent manner (**Figure 1D**).

### 3.2 Determination of hormonal, metabolic, antioxidant, and H_2_O_2_ content in *P. spadiceum* AGH786 culture filtrate

**Figure 2:**
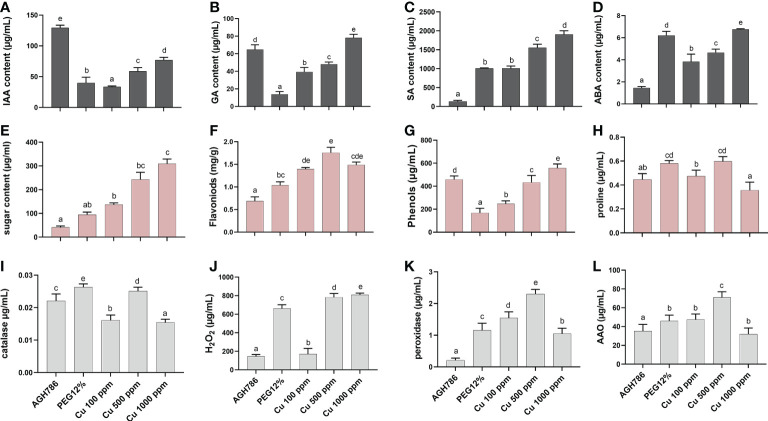
Effect of *P. spadiceum* AGH786 on hormonal content of *S. lycopersicum* L. under heavy metal (Cu) toxicity and drought stress (12% PEG). **(A)** Indole-3-acetic acid (IAA) level, **(B)** gibberellic acid (GA) level, **(C)** salicylic acid (SA) level, **(D)** abscisic acid (ABA) level, **(E)** total soluble sugars, **(F)** total flavonoids, **(G)** total phenolics, **(H)** proline content, **(I)** catalase activity, **(J)** H_2_O_2_ content, **(K)** peroxidase activity, and **(L)** ascorbate oxidase activity under different concentrations of Cu and 12% PEG in liquid media. Data represent the mean with standard error, and letters represent the significant difference (p < 0.05).

After the assessment of the tolerance response of *P. spadiceum* AGH786 against drought stress and Cu toxicity, plant growth-promoting hormones (IAA, GA, SA, and ABA) were quantified. Significantly enough of these plant growth-promoting hormones were quantified in AGH786 fungal culture growing on media (Czapek), supplemented with the different concentrations of copper salt at 100, 500, and 1,000 ppm and 12% PEG (**Figure 2**).

Moreover, hormonal contents were differentially upregulated in various concentrations of copper salt at 100, 500, and 1,000 ppm, with the highest increase in IAA (AGH786-treated media), GA (Cu 1,000 ppm), SA (Cu 1,000 ppm, followed by Cu 500 ppm), and ABA (12% PEG and Cu 1,000 ppm) upon supplementing the Cu 1,000 ppm compared with the control. However, IAA (Cu 1,000 ppm) contents were significantly (p< 0.05) decreased with an increase in SA (Cu 1,000 ppm) and ABA (Cu 1,000 ppm) contents upon supplementation of 12% PEG compared with the control culture of *P. spadiceum* AGH786 (**Figures 2A-D**).

After the assessment of the tolerance response of *P. spadiceum* AGH786 against drought stress and Cu toxicity, primary and secondary metabolites were also estimated in the *P. spadiceum* AGH786 culture filtrate grown under PEG-induced drought stress and Cu supplementation. Significantly (p< 0.05), higher concentration of soluble sugars was recorded in various concentrations of copper salt (100, 500, and 1,000 ppm), with the highest increase (Cu 1,000 ppm) upon supplementing the Cu 1,000 ppm compared with the control (**Figure 2E**).

The total soluble sugar content was also significantly increased (Cu 1,000 ppm) in *P. spadiceum* AGH786 culture grown in PEG-induced drought stress

A differentially higher concentration of total flavonoids was also recorded in various concentrations of copper salt (100, 500, and 1,000 ppm) with the highest increase (Cu 500 ppm) upon supplementing the Cu 500 ppm compared with the control. The total flavonoid content was also significantly increased in the *P. spadiceum* AGH786 culture grown in PEG-induced drought stress (**Figure 2F**).

A differentially higher concentration of total phenolics was also recorded in various concentrations of copper salt (100, 500, and 1,000 ppm) with the highest increase upon supplementing the Cu 1,000 ppm compared with the control. However, the total phenolic content was significantly decreased (12% PEG) in the *P. spadiceum* AGH786 culture grown in PEG-induced drought stress (**Figure 2G**).

The proline quantification test also showed an increase in the *P. spadiceum* AGH786 culture grown on media having various concentrations of copper salt (100, 500, and 1,000 ppm), with the highest increase upon supplementing the Cu 500 ppm compared with the control. However, the proline content was also significantly increased in the *P. spadiceum* AGH786 culture grown in PEG-induced drought stress (**Figure 2H**).

After the assessment of the tolerance response of *P. spadiceum* AGH786 against drought stress and Cu toxicity, antioxidant enzymes (CAT, POX, and AAO) and reactive oxygen species (ROS) (H_2_O_2_) were also quantified. Results showed a significant (p< 0.05) increase in POX and AAO activities detected in the *P. spadiceum* AGH786 fungal culture growing on media (Czapek), supplemented with the different concentrations of copper salt at 100, 500, and 1,000 ppm, with the highest increase in POX activity (Cu 500 ppm), AAO activity (Cu 500 ppm), and CAT activity (Cu 500 ppm) compared with the control. The H_2_O_2_ content was also increased at Cu 500 and 100 ppm supplementation compared with the control.

PEG-induced drought stress triggered a significant (p< 0.05) increase in H_2_O_2_ content and antioxidant activity of POX, CAT, AAO enzymes in the *P. spadiceum* AGH786 culture (**Figures 2I-L**).

### 3.3. Effect of *P. spadiceum* AGH786 on growth attributes of *S. lycopersicum* L. under Cu and polyethylene glycol stress

**Figure 3:**
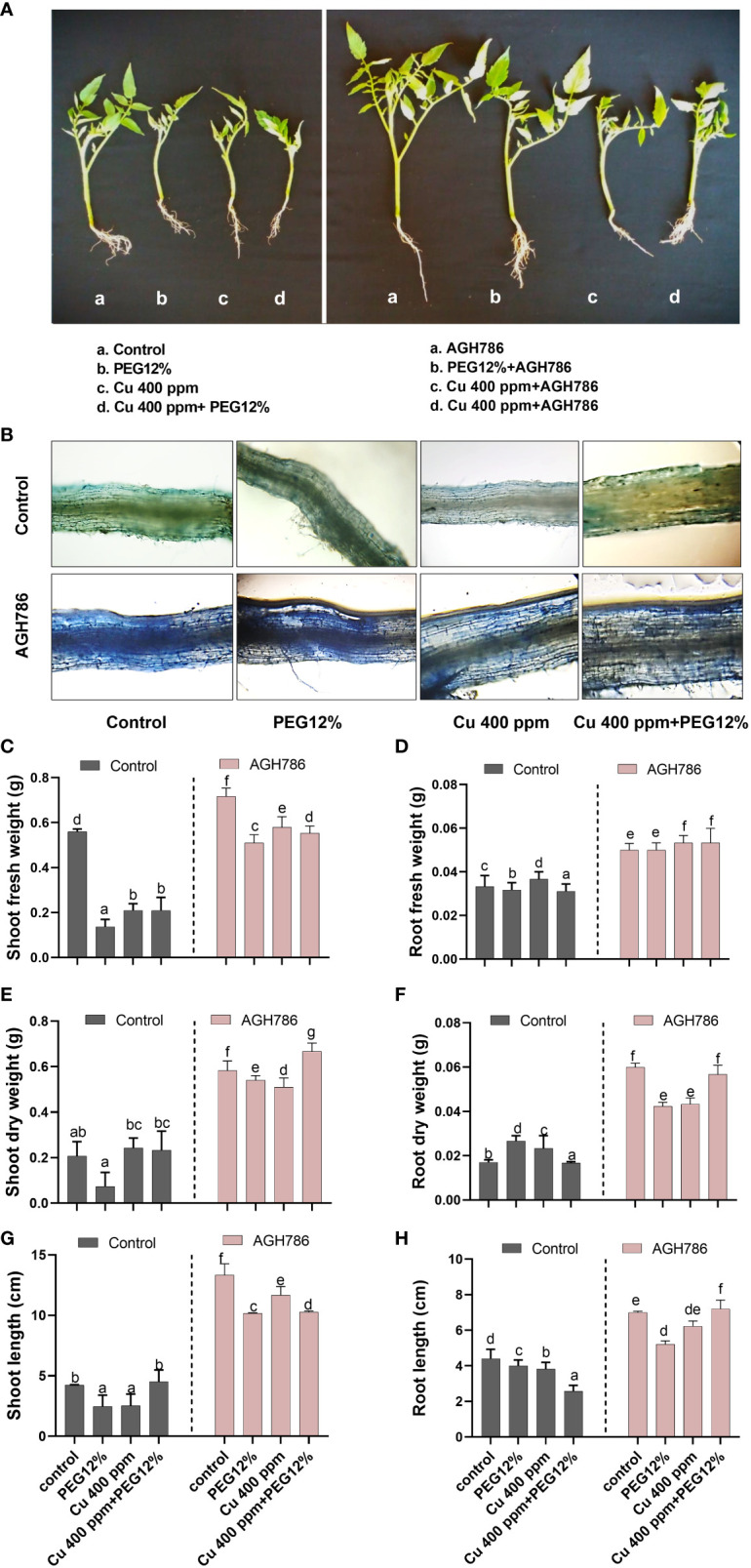
Effect of *P. spadiceum* AGH786 on growth attributes of *S. lycopersicum* L. under heavy metal (Cu) toxicity and drought stress (12% PEG). **(A)** Effects of *P. spadiceum* AGH786 on the growth of host seedlings. **(B)** Root colonization by *P. spadiceum* AGH786. **(C)** Effects of *P. spadiceum* AGH786 on shoot fresh weight. **(D)** Root fresh weight. **(E)** Shoot dry weight. **(F)** Root dry weight. **(G)** Shoot length. **(H)** Root length of host seedlings. Data represent the mean with standard error, and letters represent the significant difference (p < 0.05).

The effect of *P. spadiceum* AGH786 on *S. lycopersicum* L. plants supplemented with copper salt (400 ppm) and PEG (12%) was investigated in comparison to control, in terms of shoot–root fresh, dry weight, and shoot–root length (**Figure 3**). Root colonization by *P. spadiceum* AGH786 with *S. lycopersicum* L. was also assessed by lactophenol cotton blue staining, which confirmed the successful plant microbial interactions with the root tissue of *S. lycopersicum* L. plants under observations (**Figure 3B**).

*P. spadiceum* AGH786 inoculation significantly promoted the growth parameters in comparison to non-inoculated plants. Moreover, *S. lycopersicum* L. plants supplemented with copper salt (400 ppm) and PEG (12%) and inoculated with *P. spadiceum* AGH786 also exhibited a significant increase in the shoot length, root length, shoot fresh weight, and dry weight, compared with non-inoculated plants under stress (**Figures 3C-H**).

### 3.4 Effect of *P. spadiceum* AGH786 on photosynthetic pigments of *S. lycopersicum* L. under Cu and 12% polyethylene glycol stress

The effect of *P. spadiceum* AGH786 on *S. lycopersicum* L. plant’s photosynthetic potential, supplemented with copper salt (400 ppm) and PEG (12%), was investigated in comparison to control. The photosynthetic potential was evaluated in terms of the production of chlorophyll a and b, total chlorophyll, and carotenoids.

**Figure 4:**
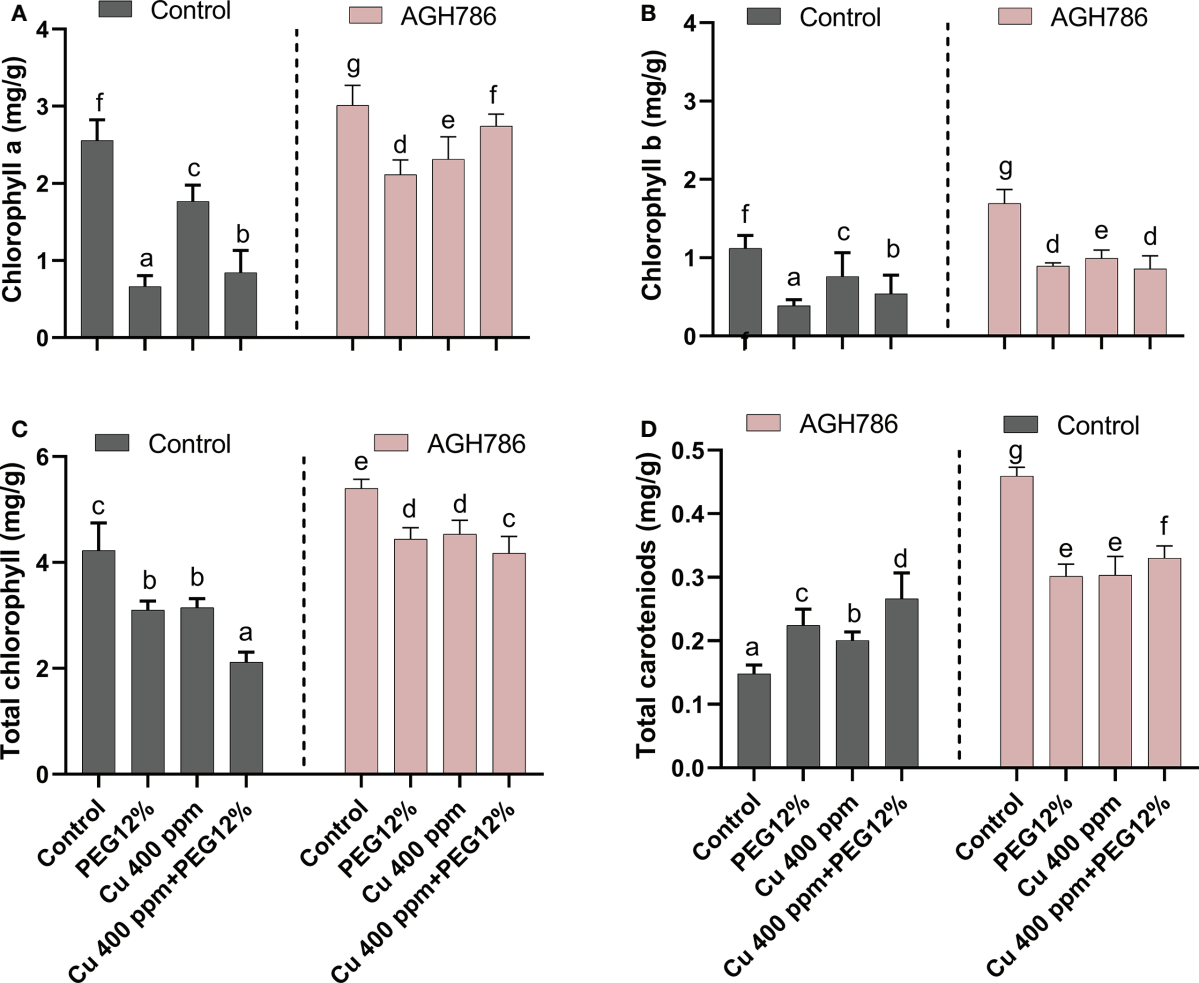
Effect of *P. spadiceum* AGH786 on the photosynthetic potential of *S. lycopersicum* L. under heavy metal (Cu) toxicity and drought stress (12% PEG). **(A)** Chlorophyll a, **(B)** chlorophyll b, **(C)** total chlorophyll, and **(D)** total carotenoids. Data represent the mean with standard error, and letters represent the significant difference (p < 0.05).

*P. spadiceum* AGH786 inoculation significantly promoted the production of chlorophyll a and b, total chlorophyll, and carotenoids in comparison to non-inoculated plants. Moreover, *S. lycopersicum* L. plants supplemented with copper salt (400 ppm) and PEG (12%) and inoculated with *P. spadiceum* AGH786 also showed a significant promotion in the production of chlorophyll a and b, total chlorophyll, and carotenoids, compared with non-inoculated plants under stress (**Figures 4A-D**).

### 3.5 Effect of *P. spadiceum* AGH786 on hormonal contents of *S. lycopersicum* L. under Cu and polyethylene glycol stress

The effect of *P. spadiceum* AGH786 on *S. lycopersicum* L. plant’s phytohormonal contents, supplemented with copper salt (400 ppm) and PEG (12%), was investigated in comparison to control.

**Figure 5:**
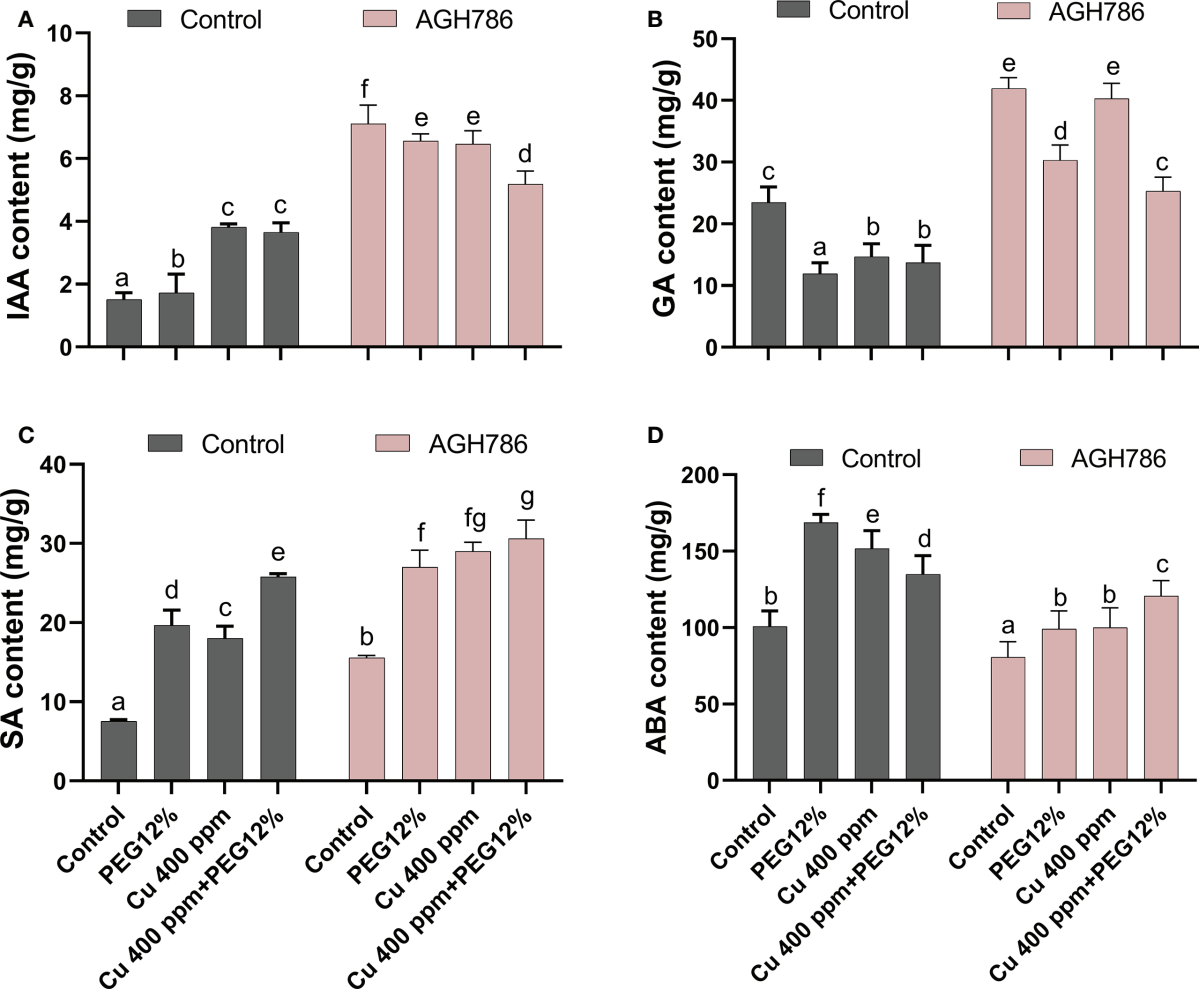
Effect of *P. spadiceum* AGH786 on hormonal content of *S. lycopersicum* L. under heavy metal (Cu) toxicity and drought stress (12% PEG). **(A)** Indole-3-acetic acid (IAA), **(B)** gibberellic acid (GA), **(C)** salicylic acid (SA), and **(D)** abscisic acid (ABA). Data represent the mean with standard error, and letters represent the significant difference (p < 0.05).

*P. spadiceum* AGH786 inoculation significantly promoted the production of IAA, GA, and SA, while a reduction in ABA levels was observed in comparison to non-inoculated plants under normal growth conditions. Moreover, *S. lycopersicum* L. plants supplemented with copper salt (400 ppm) and PEG (12%) and inoculated with the *P. spadiceum* AGH786 showed a significant promotion in the production of IAA, GA, and SA, while ABA content was also increased, compared to non-inoculated plants under stress (**Figures 5A-D**).

### 3.6. Effect of *P. spadiceum* AGH786 on metabolic attributes of *S. lycopersicum* L. under Cu and polyethylene glycol stress

The effect of *P. spadiceum* AGH786 on *S. lycopersicum* L. plant’s primary and secondary metabolic contents, supplemented with copper salt (400 ppm) and PEG (12%), was investigated in comparison to the control.

**Figure 6:**
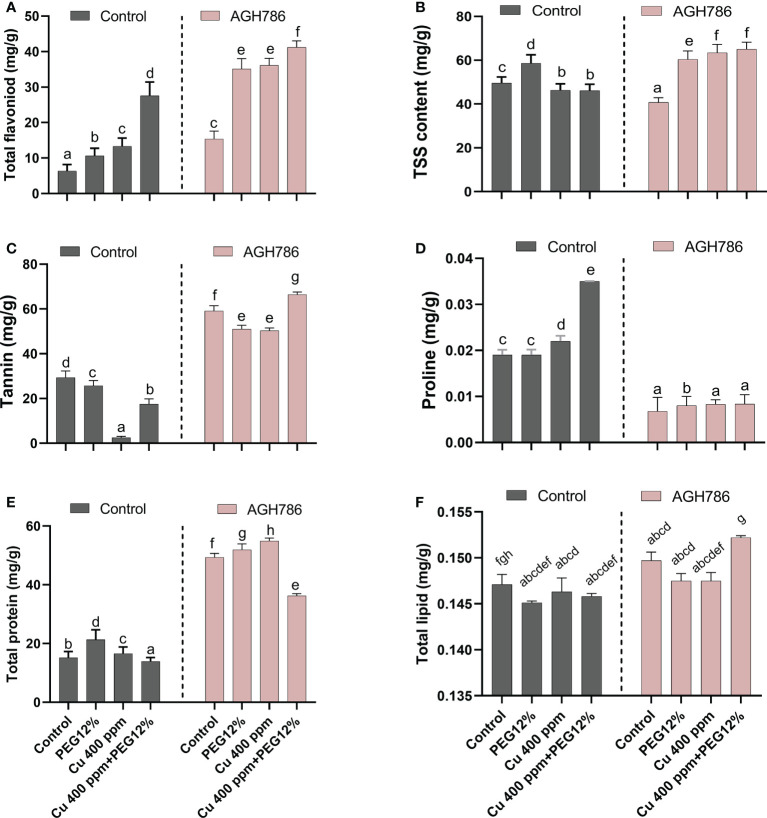
Effect of *P. spadiceum* AGH786 on metabolic attributes of *S. lycopersicum* L. under heavy metal (Cu) toxicity and drought stress (12% PEG). **(A)** Total flavonoids, **(B)** total soluble sugar, **(C)** tannins, **(D)** proline, **(E)** total protein, and **(F)** total lipids. Data represent the mean with standard error, and letters represent the significant difference (p < 0.05).

*P. spadiceum* AGH786 inoculation significantly promoted the production of total flavonoids, tannins, total proteins, and total lipids in comparison to non-inoculated plants under normal growth conditions, whereas total soluble sugar and proline levels were reduced by *P. spadiceum* AGH786 in *S. lycopersicum* L. Moreover, *S. lycopersicum* L. plants supplemented with copper salt (400 ppm) and PEG (12%) and inoculated with *P. spadiceum* AGH786 showed a significant promotion in the total flavonoids, tannins, total proteins, total soluble sugar, and total lipids in comparison to non-inoculated plants under stress, whereas proline level was reduced by *P. spadiceum* AGH786 in *S. lycopersicum* L. compared with non-inoculated plants under stress (**Figures 6A-F**).

### 3.7 Effect of *P. spadiceum* AGH786 on reactive oxygen species generation and antioxidant potential of *S. lycopersicum* L. under Cu and polyethylene glycol stress

**Figure 7:**
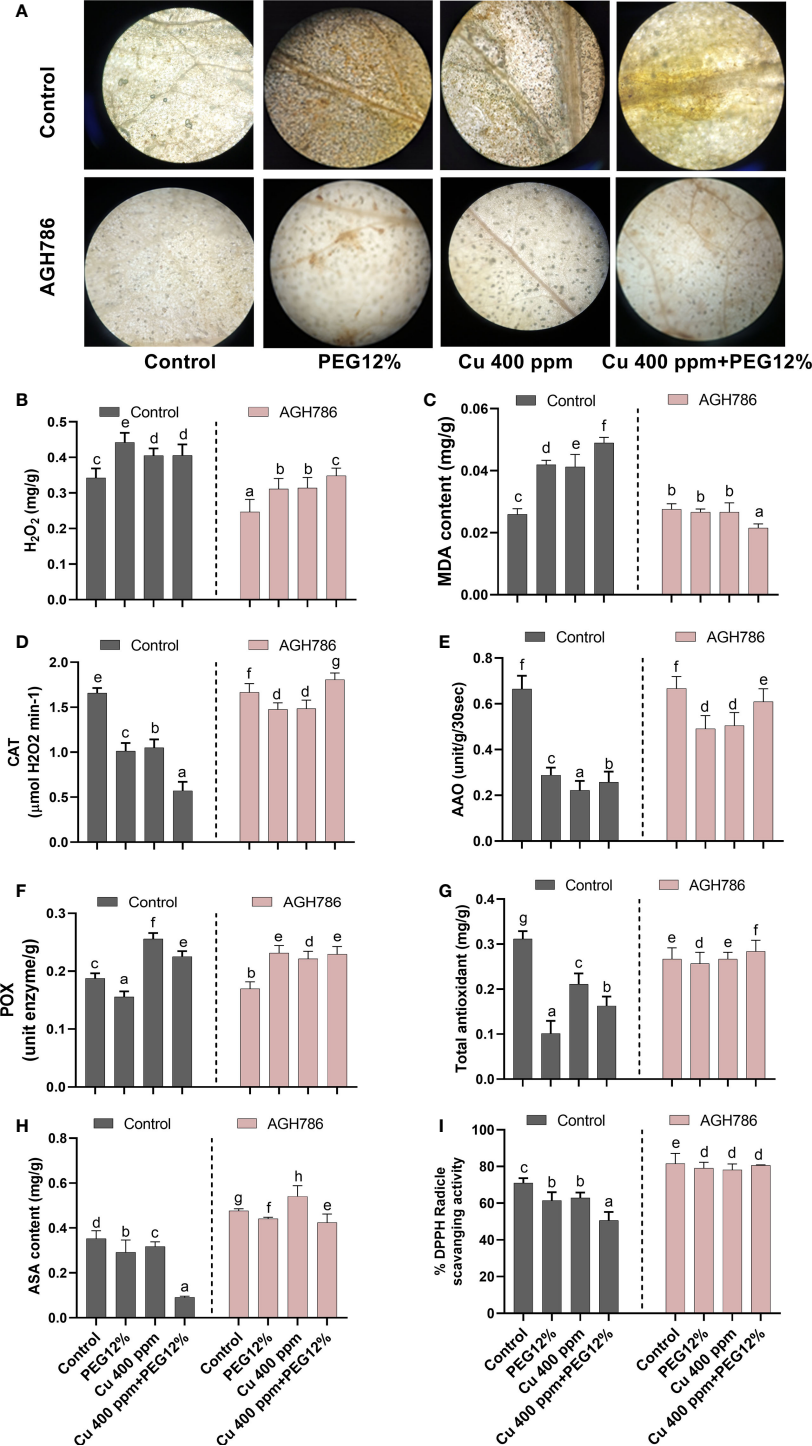
Effect of *P. spadiceum* AGH786 on the antioxidant potential of *S. lycopersicum* L. under heavy metal (Cu) toxicity and drought stress (12% PEG). **(A)** Effects of AGH786 on endogenous ROS accumulation. **(B)** H_2_O_2_ level. **(C)** MDA content. **(D)** Catalase activity. **(E)** Ascorbate oxidase activity. **(F)** Peroxidase activity. **(G)** Total antioxidants. **(H)** Ascorbic acid. **(I)** DPPH activity. Data represent the mean with standard error, and letters represent the significant difference (p < 0.05).

In response to heavy metal toxicity and drought stress, oxidative damage response in terms of ROS production was evaluated in *S. lycopersicum* L. upon inoculation of *P. spadiceum* AGH786. To this end, the amount of H_2_O_2_ was observed as brown spots by using DAB staining in the leaves of *S. lycopersicum* L. (**Figure 7A**). A higher amount of H_2_O_2_ accumulation was recorded in the individual treatment of Cu and 12% PEG in plant tissues, whereas the highest increase was found in plants treated with the combined treatment of Cu and 12% PEG, in comparison to control. The inoculation of *P. spadiceum* AGH786 induced a reduction in ROS production and H_2_O_2_ accumulation in plants under stress compared with the non-inoculated control (**Figures 7A, B**).

MDA content (product of lipid peroxidation in biomembranes degradation by ROS overproduction) was quantified in *S. lycopersicum* L. plants under stress upon inoculation of *P. spadiceum* AGH786. Results showed that *S. lycopersicum* L. plants under stress upon inoculation of *P. spadiceum* AGH786 exhibited lower MDA content compared with the non-inoculated control (**Figure 7C**).

The effect of *P. spadiceum* AGH786 on *S. lycopersicum* L. plant’s antioxidant potential, supplemented with copper salt (400 ppm) and PEG (12%), was investigated in comparison to the control. Antioxidant potential was evaluated in terms of induction of enzymatic (CAT, POX, and AAO) and non-enzymatic antioxidants (AsA), free radical scavenging activity, and total antioxidant production.

*P. spadiceum* AGH786 inoculation significantly induced the enzymatic (CAT, POX, and AAO) and non-enzymatic antioxidants (AsA) of *S. lycopersicum* L. plants in comparison to non-inoculated plants. Moreover, *S. lycopersicum* L. plants supplemented with copper salt (400 ppm) and PEG (12%) and inoculated with *P. spadiceum* AGH786 also showed significant induction in the enzymatic (CAT, POX, and AAO) and non-enzymatic antioxidants (AsA), compared with non-inoculated plants under stress (**Figures 7D–I**).

### 3.8 Effects of *P. spadiceum* AGH786 on heavy metal Cu uptake in *S. lycopersicum* L. under normal and drought stress

**Figure 8:**
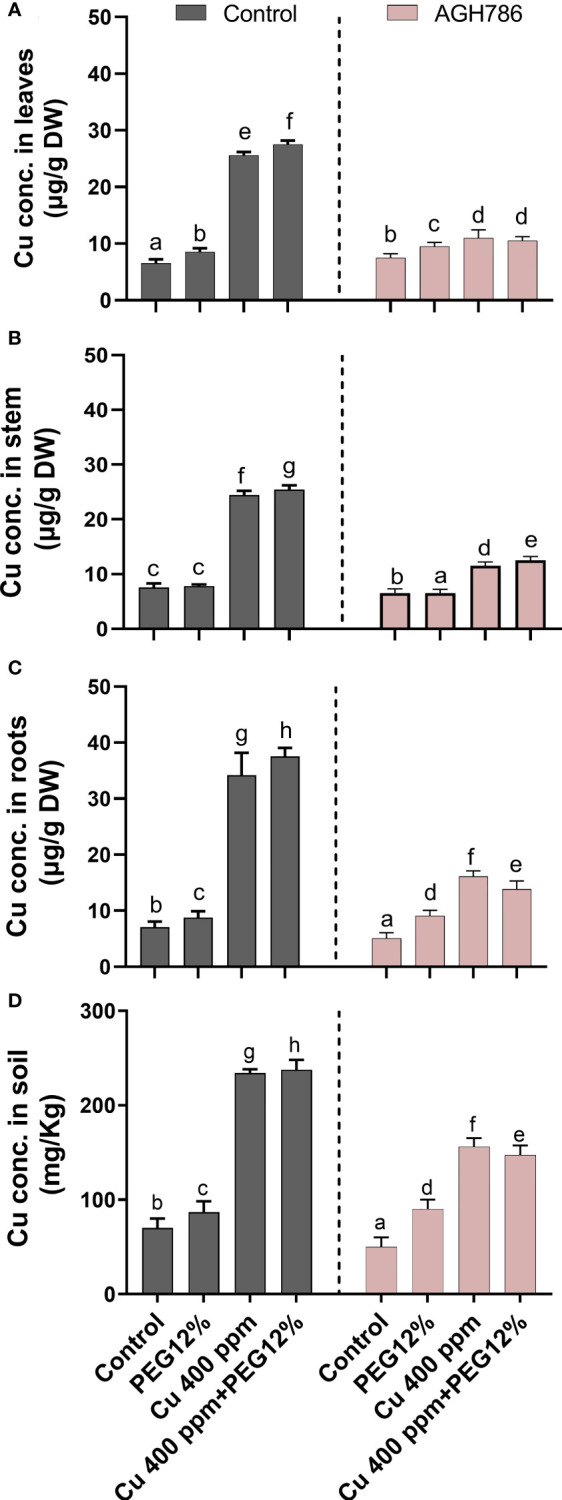
Effect of *P. spadiceum* AGH786 on the endogenous copper accumulation in various tissues of *S. lycopersicum* L. under heavy metal (Cu) toxicity and drought stress (12% PEG). **(A)** Cu accumulation in leaves. **(B)** Cu concentration in the stem. **(C)** Cu concentration in roots. **(D)** Concentration of Cu in soil. Data represent the mean with standard error, and letters represent the significant difference (p < 0.05).

*P. spadiceum* AGH786 decreased the toxicity of Cu through limited uptake, translocation, and accumulation in the upper parts of the *S. lycopersicum* plants (**Figure 8**). A significant reduction in Cu was found in the leaves of *S. lycopersicum* associated with *P. spadiceum* AGH786 as compared with the stem and root tissues of inoculated and non-inoculated plants under combined stress of heavy metal (Cu) and drought stress (12% PEG). Significantly higher Cu content was retained in the soil pots with *S. lycopersicum* L. plants associated with the *P. spadiceum* AGH786 fungal endophyte compared with the non-inoculated control indicating a reduction in the uptake of the Cu content from the soil by root tissue of plants under Cu stress, as shown in **Figures 8C, D**.

However, an opposite trend was recorded in the plants treated with Cu, which showed an abrupt increase in the uptake of Cu by root tissues and overaccumulation in the shoot tissue, whereas the highest Cu content was quantified in the leaf tissues of the non-inoculated *S. lycopersicum* plants under Cu stress compared with the control. Significantly low Cu content was retained in the soil pots with non-inoculated *S. lycopersicum* L. plants compared with the inoculated control, indicating a sufficient uptake of Cu to the root tissues of the plants under Cu and drought stress.

### 3.9. Effects of AGH786 on *Cu^2+^ transporters* and *metallothioneins* gene expressions in *S. lycopersicum* L. under stress

The RT-qPCR analysis was carried out to evaluate the effect of AGH786 inoculation on the expression level of selected *Cu^2+^ transporters (COPTs)* and *MTs* genes in the leaf and root tissues of *S. lycopersicum* L. plants grown under single and combined Cu and drought stress.

**Figure 9:**
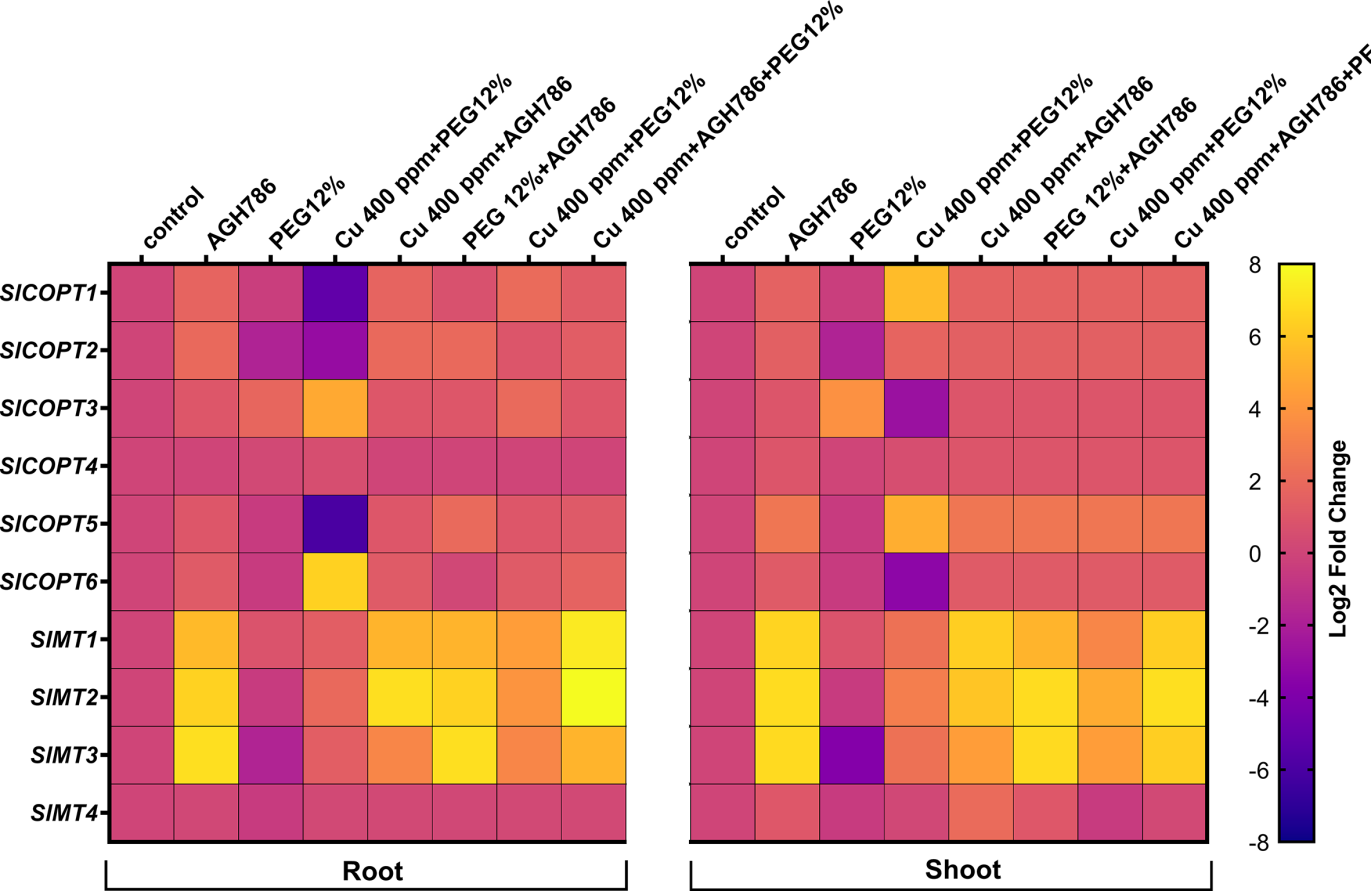
Differential expression profile of *SlCOPT* and *SlMT* genes by Reverse transcription–quantitative PCR in the root and shoot tissues of *S. lycopersicum* L. subjected to the single and combined stress of Cu and drought inoculated with AGH786 compared with the control. Quantitative data represent the means ± standard deviation of three independent experiments and at least three technical replicates each.

The expression levels of the *SlCOPT1*, *SlCOPT2*, *SlCOPT3*, *SlCOPT4*, *SlCOPT5*, and *SlCOPT6* and *SlMT1, SlMT2, SlMT3*, and *SlMT4* in the roots and leaf tissues of *S. lycopersicum* L. are shown in **Figure 9**. The analysis revealed that *SlCOPT3* and *SlCOPT6* genes exhibited a significantly higher expression (>4-fold), both in root and shoot tissue, in response to Cu stress. However, AGH786 inoculation significantly decreased the expression of *SlCOPT3* and *SlCOPT6* genes up to the basal level, both in root and shoot tissues of *S. lycopersicum* L. under single and combined stress of Cu and drought (**Figure 9**).

The expression levels of *SlMT1, SlMT2, SlMT3*, and *SlMT4* in the roots and leaf tissues of *S. lycopersicum* L. are shown in **Figure 9**. The analysis revealed that *SlMT1, SlMT2*, and *SlMT3* genes exhibited a significantly higher expression in leaf and root tissues (>4-fold change) in response to Cu stress, whereas expression was downregulated (>0.5 fold) in response to drought stress in root and shoot tissues. AGH786 inoculation induced the expression of *SlMT1, SlMT2*, and *SlMT3* genes (>5-fold) both in root and shoot tissues of *S. lycopersicum* L. Moreover, the combined stress of Cu and drought was induced (>3-fold), which was further increased (>6-fold) by AGH786 inoculation in *S. lycopersicum* L. plants (**Figure 9**).

## 4 Discussion

In general, microorganisms exhibit high tolerance to multiple stresses (drought and heavy metals), acquired likely through an evolutionary adaptation to a contaminated, harsh environment. Fungi are more tolerant to environmental heavy metals (HMs) than other microorganisms, for instance, bacteria, because of differences in cellular metabolism (Rajapaksha et al., 2004; Agustinho et al., 2018). Higher osmotic pressure in the cell structure of fungi allows them to survive adverse conditions (Agustinho et al., 2018). Moreover, fungi can survive in the soil as sclerotia, chlamydospores, or other structures that allow the microorganisms to survive under unfavorable conditions (Golubović-Ćurguz 2010).

High tolerance of fungi has been observed when the tolerance thresholds to Cu of pure cultures of systematically distant soil microorganisms were compared. At high Cu concentrations (128 mmol kg^−1^) applied to growing media, fungal activity (acetate-in-ergosterol incorporation rate) increased by seven times as compared with the control (Rajapaksha et al., 2004).

Most of the plant growth-promoting endophytic fungi belong to the group of sac fungi known as *Ascomycota*. However, members of club fungi (*Basidiomycota*) have also been shown to exist as endophytes in plant tissues and promote growth by different mechanisms (Waller et al., 2005; Khan et al., 2009).

Several *Ascomycota* filamentous fungi have been known to be heavy metal stress resistant. For example, the *Rhizopus microsporus* was found highly tolerant to a wide range of Cu concentrations (400–1,000 mg kg^−1^); however, its high tolerance capacity was apparent only at 25 mg kg^−1^ of Cd and 125 mg kg^−1^ of arsenic (As) (Oladipo et al., 2018). Organic acids induced tolerance to copper-exposed filamentous fungi (*A. niger* and *P. citrinum*) (Sazanova et al., 2015).

However, among *Basidiomycota*, only a few members have been reported to be heavy metal stress tolerant. For example, white rot basidiomycetes *Abortiporus biennis* and *C. unicolor* showed a species-specific response to Cd stress. Cd biosorption onto the mycelial surface was the predominant Cd sequestration mechanism in *C. unicolor* that induced the Cd stress tolerance of *C. unicolor* in comparison to *A. biennis* (Cd-sensitive). These species-specific responses toward Cd suggest that *C. unicolor* possesses a more efficient system than *A. biennis* to keep intracellular Cd concentrations low. *A. biennis* showed higher content of thiol compounds (cysteine, *γ*-glutamylcysteine, and glutathione in both its reduced and oxidized form) by Cd application, whereas *C. unicolor* showed higher production of oxalate and laccase by Cd application, which is corroborated by the Cd stress tolerance response of *C. unicolor* (Jarosz-Wilkołazka, 2006). Oxalic acid overproduction also triggered Cu toxicity tolerance in brown rot basidiomycete fungi *(P. placenta, M. incrassate, W. cocos*, and *A. vaillantii*) (Clausen and Green (2003). Reddy et al. (2014) found differential expression of MTs in response to heavy metals and their involvement in metal tolerance in the symbiotic basidiomycete *L. bicolor*. Combining plants and their associated microorganisms to eliminate contaminants and provide environmental stress alleviation provides a cost-effective, *in situ*, and promising technology (Tiodar et al., 2021).

Moreover, root-associated microorganisms, such as mycorrhizal fungi and endophytic fungi, can remove, inactivate, or degrade harmful environmental contaminants (Aziz et al., 2021a; Aziz et al., 2021b; Wang et al., 2022). Endophytic fungi are the essential components of root microflora in the metal-contaminated ecosystem. They possess various degradation pathways by which they increase host heavy metal tolerance and assist the host’s survival in contaminated soils, for example, extracellular metal sequestration (by secreting organic acids and compounds), metal binding to cell walls (hydroxyl, amide, carboxyl, and phosphate-rich cell walls of the lignin-degrading fungus), intracellular metal immobilization (through metal transporters and efflux pumps), and chemical transformations and compartmentalization (through metal chelators) (Gajewska et al., 2022).

During symbiosis, endophytic fungi either directly induce resistance of the host plants to deal with heavy metal toxicity as “phytoremediators” or indirectly improve tolerance by improving water and mineral nutrient uptake in plants, increasing shoot biomass and causing modification in the root morphology. In addition to their ability to promote plant growth, endophytes can chelate and/or sequester heavy metals in polluted soil (Zahoor et al., 2017). Therefore, these are called “mycoremediators.” In addition, endophytic fungi–assisted phytoremediation is a cost-effective and environmentally friendly strategy (Wani et al., 2015).

Only a few reports of endophytic fungal members of *Basidiomycota* are there, such as *P. indica*, that could improve the tolerance of host plants to heavy metals that immobilized the heavy metals in host plant roots, which can be very promising in phytoremediation (Shahabivand et al., 2017; Ghorbani et al., 2020).

Endophytic fungus *P. spadiceum* AGH786 (a member of *Basidiomycota*) isolated from the roots of soybean (cv. Hwangkeumkong) by Hamayun et al. (2017) demonstrated resistance to drought and Cu stress and induced combined stress tolerance against drought and Cu in *S. lycopersicum* L. by colonizing the roots of host plants under stress.

Sessile plants are permanently confined to their germination place. Some plant species have adapted growth responses (morphological, physiological, biochemical, and molecular adaptations) to deal with the profuse and quick variations in environmental stress, such as drought, through diversity in the context of stress adaptation, higher plants develop sophisticated abiotic stress responses too, such as resistance to drought, to optimize growth under stress (Takahashi et al., 2020). ABA is known as a stress hormone that responds to stress conditions like drought by closing its stomata and expressing stress-related genes (Cutler et al., 2010). In the scarcity of water, ABA accumulates in leaf vasculature because of the response to drought stress. ABA biosynthesis occurs in leaf vasculature tissues (Takahashi et al., 2018).

Unfortunately, not all plant species have the capacity to adapt to the changing environment for their survival and growth under stress. Researchers have found that endophytic fungi directly or indirectly induce the resistance of the host plants against various biotic and abiotic stresses (Rauf et al., 2021; Javed et al., 2022; Rauf et al., 2022) to deal with water stresses by improving water and mineral nutrient uptake, modulating antioxidant capacity to cope with ROS-prone destruction upon stress in plants. Endophytic fungi–assisted drought stress alleviation is a cost-effective and environmentally friendly strategy.

Endophytic fungi have also been found to secrete large amounts of secondary metabolites such as terpenoids, alkaloids, phenalenones, cytochalasins, terphenyls, xanthones, diphenyl ether, sterols, squalene, gliotoxins, and their derivatives with varied biological functions (El-Hawary et al., 2020; Rauf et al., 2022).

In this study, *P. spadiceum* AGH786 ably tolerated with a normal growth response on media supplemented with different concentrations of Cu from 100 to 1,000 ppm. Moreover, the growth response of *P. spadiceum* AGH786 was equally normal upon induced drought 12% PEG compared with the control. These findings support the hypothesis that *P. spadiceum* AGH786 is a multistress-tolerant endophytic fungus that can be exploited for growth promotion and induction of multistress resistance in *S. lycopersicum* L.

Current research also shows that *P. spadiceum* AGH786 has a strong potential for producing and secreting primary and secondary metabolites and growth hormones such as IAA, GA, SA, ABA, flavonoids, phenolics, sugar, and proline. The sufficiently produced growth-related metabolites and hormones consistently supported the positive role of *P. spadiceum* AGH786 as a growth-promoting endophytic fungus. Moreover, *P. spadiceum* AGH786 also produced enough enzymatic antioxidants (CAT and AAO), both under PEG-induced drought and Cu stress (in a dose-dependent differential manner).

It is known that ROS are the metabolic byproduct of photosynthesis and respiration that upon overproduction have the potential to cause oxidative damage to cells during environmental stresses. However, ROS play a key role in plants as signal transduction molecules involved in mediating responses to environmental stresses and different stimuli for growth and development. The basal antioxidant system of the cell helps to mediate the ROS overaccumulation by scavenging activities (Tudzynski et al., 2012). Consistent with previous reports, higher H_2_O_2_ accumulation in the culture filtrate of *P. spadiceum* AGH786 grown in media supplemented with 12% PEG and Cu (100–1,000 ppm) compared with the control can be explained by the activation of signaling mechanisms to support fungus growth and stress responses. The scenario may be suitable for stress alleviation in associated host plants with H_2_O_2_ produced by endophytic fungus tended to induce the antioxidant machinery of not only fungal cells but also for plant tissues resided by endophytic fungus.

In the current situation, *P. spadiceum* AGH786 inoculation to *S. lycopersicum* L. and heavy metal and induced drought stress proline content were positively regulated in non-inoculated plants, whereas in the same condition, lipid content was negatively regulated under copper and 12% PEG stressed environments. This is the reason that proline acts as an osmolyte, direct free radical (ROS) scavenger, as well as normalizes intracellular redox homeostasis.

In addition, plants can respond rapidly to water imbalance (drought) by accumulating various osmolytes like proline Many plants have been shown to accumulate proline in large quantities when exposed to heavy metal stress. However, despite its beneficial effects, proline may be toxic if overaccumulated or applied in excessive concentrations (Mostofa et al., 2015). In this work, we found that Cu stress induced a high increase in the proline level, whereas AGH786 inoculation to *S. lycopersicum* moderately reduced the accumulated proline content to a moderate level and induced the tolerance in the seedlings under single and combined Cu and drought stress.

Several reports have shown that ROS has the potential to cause oxidative damage to cells during environmental stresses. However, ROS plays a key role in plants as signal transduction molecules involved in mediating responses to environmental stresses, programmed cell death, and different developmental stimuli (Mittler et al., 2004; Torres and Dangl 2005). The rapid increase in ROS production is referred to as “the oxidative burst.”

In our study, it was found that *S. lycopersicum* L. exposed to abiotic stresses such as copper and induced drought 12% PEG reduced host growth by slowing down their metabolic activities. *P. spadiceum* AGH786 inoculation enhanced and stimulated the growth of the host plant under combined stress of drought and Cu, helped to detoxify copper metal by restricting the Cu uptake by roots and sequestrating the excessive amount in roots by metal chelators, and adapted to induced drought conditions by strengthening osmolyte production and enhancing the antioxidant potential of *S lycopersicum* L.

Consistent with the previous studies, the *P. spadiceum* AGH786 endophytic fungus also modulated the hyperactivity of various antioxidants (CAT, AAO, POD, and DPPH) in *S. lycopersicum* L., which primarily helped to scavenge the overproduced ROS under the combined stress of Cu toxicity and drought (Evelin et al., 2019; El-Esawi et al., 2020). Among the antioxidant enzymes’ catalytic activity by converting the molecules of H_2_O_2_ into simple molecules of water and oxygen, ascorbate peroxidases (AAO) convert H_2_O_2_ into H_2_O and use it as an electron donor. POD oxidizes aromatic electron donors such as guaiacol and pyrogallol at the expense of H_2_O_2_ (Engwa, 2018). The present research also demonstrated the ability of *P. spadiceum* AGH786 to associate with *S. lycopersicum* L. seedlings and the potential of quenching DPPH to reduce the accumulation of free radical ROS.

Previous reports have shown that MDA is overproduced upon stress in plants because of the cellular destructive activities of ROS (Hasanuzzaman et al., 2020). As a result, endophytic fungi (*P. spadiceum*, AGH786) in the current study help *S. lycopersicum* produce enough antioxidant enzymes to stop MDA production and detoxify the cells from ROS by scavenging the overproduced free radicals in the stressed host.

Although the Earth crust is made up of natural heavy metal elements, their proportion has been altered by anthropogenic activities such as rapid industrialization, extensive irrigation systems, and agricultural practices. Involuntarily, these heavy metals enter the food chain through overabsorption or accumulation by growing crop plants in contaminated soils. The overaccumulation of these heavy metals in plants decreases plant growth. In such conditions, bioremediation techniques (including mycoremediation and phytoremediation) are useful as compared with other approaches (Aziz et al., 2021a). Our results showed that *P. spadiceum* AGH786 is a growth-promoting endophytic fungus. Inoculation of *S. lycopersicum* L., along with copper stress and induced drought stress, relieved copper toxicity and reduced induced drought effects on host plants through biochemical, physiological, and molecular strategies.

Our results also revealed the positive role of *P. spadiceum* AGH786 in helping in restricting Cu uptake by roots and translocation of Cu from root to shoot. Thereby, copper accumulation in roots, stems, and leaf tissues was predominantly less than the toxic level for host plants, compared with non-inoculated *S. lycopersicum* L.

Root-to-shoot translocation is a crucial activity for plants that is an important limiting factor for the transportation of the soil resources up to the fruits. A current study consistently showed that *P. spadiceum* AGH786 association helped the plant to prevent copper metal transport to leaves and other upper parts like stems and leaves of the host plant during the vegetative stage of plant growth. The roots of plants have direct contact with soil, and all types of toxic metal ions affect the roots directly (Shahabivand et al., 2016). Increased accumulation of heavy metals in roots and their translocation to the upper aerial part are observed in *S. lycopersicum* L. without the *P. spadiceum* AGH786 association. These findings indicated the potential of *P. spadiceum* AGH786 to remediate the excessive Cu ions in the soil, as well as the roots of the host plant, by restricting the uptake through plant root–localized Cu transporter channels. Moreover, since *P. spadiceum* AGH786 can take up and accumulate Cu content in its biomass, most of the Cu content from the soil is probably eliminated by the mycoremidiation activity of the *P. spadiceum* AGH786 fungus. Previously, the role of fungal endophytes has also been identified to restrict these heavy metals outside the roots through extracellular absorption mechanisms, and the huge accumulation of these metals in the root endodermis in casparin strips blocks the translocation of metal to the leaves (Li et al., 2014).

Fungal endophytes have also evolved various ways to eliminate the heavy metal contents from soil and the host plants directly or indirectly, such as *Lindgomycetaceae* P87 and *Curvularia geniculata* P1, which were found to reduce mercury ion Hg (II), and the reaction led to the formation of volatile forms of Hg enabling its evaporation (Pietro-Souza et al., 2020). However*, A. flavus–associated* tomato plants developed tolerance against Cd and Cr toxicity *via* the expression of *SlGSH1* and *SlPCS1* genes. Both genes helped in metal chelation and mitigated Cd and Cr toxicity. Previously, the overexpressions of *GSH1, GSH2, PCS1*, and *PCS2* (Gasic and Korban, 2007; Kühnlenz et al., 2014) were also shown to increase heavy metal tolerance by raising glutathione (GSH) and phytochelatin (PCs) levels. In addition, metal-tolerant proteins (MTPs) are divalent cation transporters and play fundamental roles in plant metal tolerance and ion homeostasis. The expression patterns of cucumber MTP genes under Zn^2+^, Cu^2+^, Mn^2+^, and Cd^2+^ stress have been studied where these MTPs were induced by a metal ion, suggesting their involvement in metal tolerance or transportation (Jiang et al., 2022).

Several genes have also been reported to be upregulated by Cu excess, including laccase-like multicopper oxidases (Berni et al., 2019). They oxidize Cu (I) to a less toxic Cu (II). The genes upregulated by Cu excess also include *Cu2+ transporters (COPT), a Cu2+ transporting P-type ATPase (HMA5)*, or two Cu chaperones (*antioxidant protein1; ATX1* and *ATX1-like Cu chaperone*) and *copper-modified resistance1 (cmr1)* protein (Puig and Thiele, 2002; Sancenón et al., 2004; Andrés-Colás et al., 2006; Juraniec et al., 2012; Shin et al., 2012). The Cu chaperones, *antioxidant protein1 (ATX1)* family of Cu chaperones specifically deliver Cu to heavy metal P-type ATPases. The *Arabidopsis thaliana* expresses the *ATX1-like Cu chaperone CCH*, which exhibits a plant-specific *carboxy-terminal domain* with unique structural properties (Andrés-Colás et al., 2006).

It is also known that non-Cu accumulator plants store excess Cu in S-rich MTs, as suggested by Mijovilovich et al. (2009) that control heavy metal homeostasis and attenuate heavy metal–induced cytotoxicity by chelation, thus lowering their intracellular concentrations. Therefore, MTs have been used as bimolecular markers for evaluating metal toxicity response indicators within plants and environmental pollution in the soil.

In this study, to reveal the uptake, transport, and accumulation of mineral elements in *S. lycopersicum* L., it was inevitable to quantify the endogenous mineral concentration and biomarker gene expression analysis induced or fluctuated by the perturbance. A six-member family of *COPT (SlCOPT1-6)* was identified and characterized in *S. lycopersicum* L. that are known to play important roles in Cu homeostasis, including absorption, transportation, and growth in plants. Furthermore, all the *SlCOPTs* contained several Cu-responsive elements (*CuRE, GTAC motif*) and different types of *cis*-elements related to hormone response, in which those related to ABA predominated. The responsive elements associated with ABA, cytokinins, GA, and auxin were found in all the *SlCOPT* members (Romero et al., 2021), indicating the induction of *SlCOPT under the control of Cu and hormonal signaling.*

It is also known that non-Cu accumulator plants store excess Cu in S-rich MT-type structures, as suggested by Mijovilovich et al. (2009). Plant MTs are thought to have a functional role in heavy metal homeostasis, and they are used as biomarkers for evaluating environmental pollution. MTs have low molecular weight (7–9 kDa), are cysteine-rich, and possess high affinity for heavy-metal, stress-responsive proteins. Different expressions of *MTs* may be linked to their biochemical and physiological functions. Additionally, MTs act as chelators of heavy metals. They are essential for metal homeostasis and detoxification, and they have important functions in the elimination of intracellular free radicals. In addition, the thiol groups in MTs can act as powerful antioxidants, so MTs may have a role in protecting against oxidative damage. *MT* expression is tissue specific and under developmental control, and several key plant hormones can play a prominent role in the regulation of the *MT* gene expression.

Previously, it was also reported that the *SlMT* genes showed a differential expression pattern when exposed to some heavy metals such as Cu, Zn, and Fe (Ryan et al., 2013). The expression of *SlMT3* was induced in roots, leaves, and fruits exposed to Cu compared with untreated groups, and *SlMT4* was significantly increased in fruits of *S. lycopersicum* L. exposed to Cu and 12% PEG. Although Cu and applications have increased *SlMT1* and *SlMT2* gene expressions compared with the control in all tissues of *S. lycopersicum* L. subjected to different concentrations of heavy metals, the highest levels of *SlMT1* and *SlMT2* transcripts were found in roots and leaves, respectively (Ryan et al., 2013). We also aimed to evaluate the expression of biomolecular marker *SlMT* genes (*SlMT1*, *SlMT2*, and *SlMT3*) in plants exposed to single and combined copper and drought stress. Consistently, this study also revealed the differential expression of *SlMT1, SlMT2*, and *SlMT3* induced in *S. lycopersicum* L. plants under single and combined Cu and drought stress inoculated with the *P. spadiceum* AGH786 endophytic fungus. From current findings, we concluded that AGH786 appeared as an efficient *S. lycopersicum* L. growth-promoting and multistress-alleviating endophytic fungus, and hence, it can be used as a biofertilizer in heavy metal–contaminated fields to rescue the crops under combined stress of Cu toxicity and drought.

## 5 Conclusion

**Figure 10:**
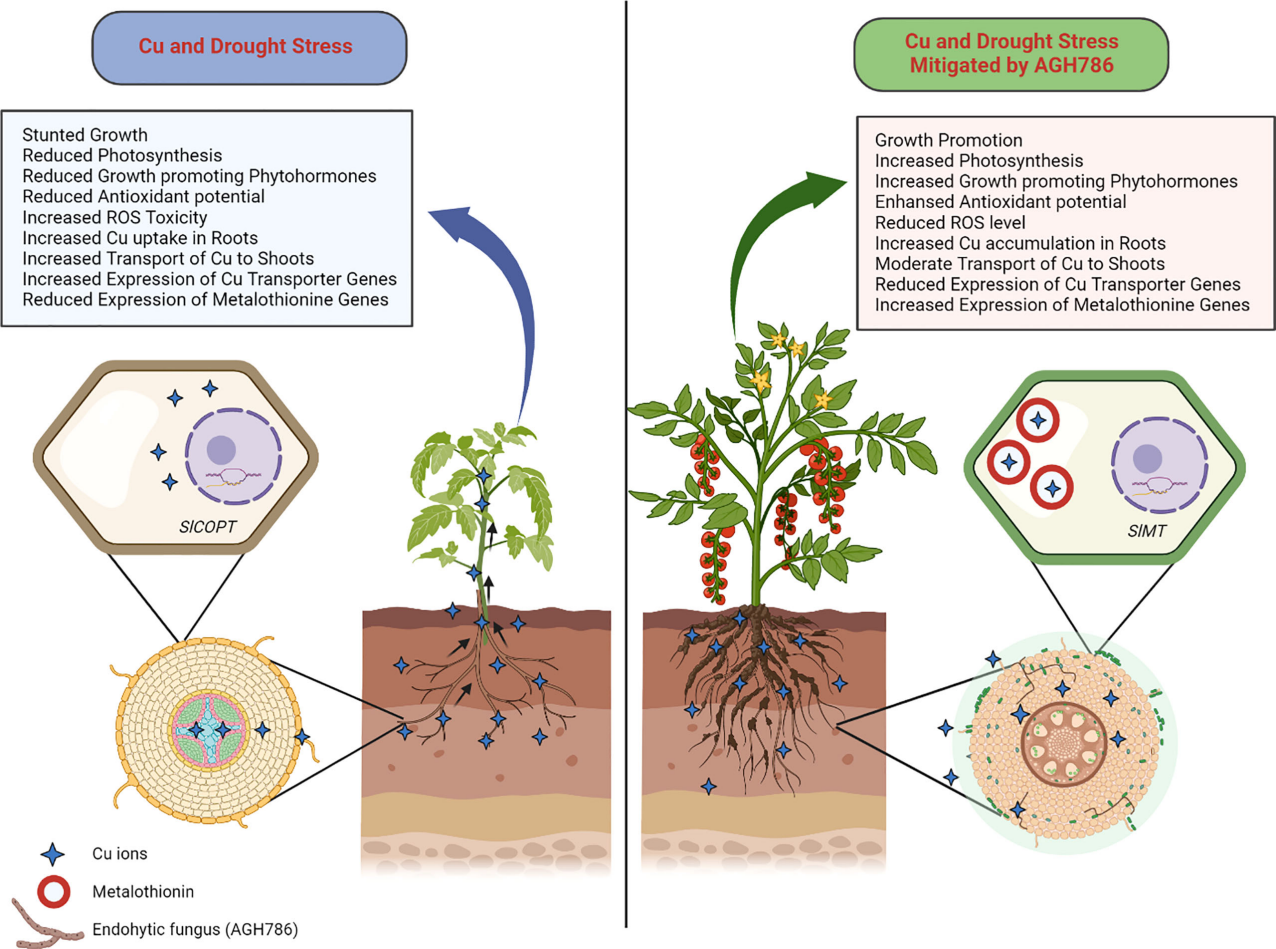
Role of *P. spadiceum* AGH786 under combined stress of Cu and drought in *S. lycopersicum* L. Cu and drought stress inhibited plant growth, while the association of AGH786 ameliorated *S lycopersicum* L. growth under Cu and drought stresses, through secreting phytohormones and essential secondary metabolites under stress conditions and modulating the plant gene expression of Cu transporters, metal chelators, and stress-related biomarker genes such as the *SlCOPT* and *SlMT* genes in the *S. lycopersicum* L. for restricting and sequestering the heavy metal ions in the root tissue.

Based on the outcomes of this study, it can be stated that the plant growth-promoting endophytic fungus *P. spadiceum* AGH786 is a multistress-resistant isolate that not only eliminated the Cu contamination from the soil through mycoremediation but also triggered the plants’ defense mechanism to cope with Cu toxicity. Moreover, the *P. spadiceum *AGH786 fungal association also boosted the signaling mechanism of host plants to modulate and optimally suppress the Cu uptake and translocation machinery and enhance the toxic metal chelation mechanism in roots, thus hindering the Cu uptake from roots and transport to upper vegetative parts and converting the host plants into efficient phytoremediators for Cu-contaminated soils (**Figure 10**).

Moreover, being drought resistant, the *P. spadiceum AGH786* isolate efficiently induced the resistance of host plants against PEG-induced drought stress. In addition to this, the *P. spadiceum *AGH786 isolate efficiently induced soil-related multistress tolerance in host plants against drought as well as Cu contamination.

## Data availability statement

The raw data supporting the conclusions of this article will be made available by the authors, without undue reservation.

## Author contributions

MH conceived the idea and designed the experiments. FN performed the main experiments. FN, MR, and MH prepared the manuscript. FN, MR, and MA analyzed the data. FN and SAK performed heavy metal content analysis. JU, MA, and MR performed the qRT-PCR analysis. HG, AH, AI, and I-JL reviewed the manuscript critically. MH, MA, H-YK, and I-JL provided financial support. All authors contributed to the article and approved the submitted version.

## Funding

The authors declare that the current research was supported by a National Research Foundation of Korea grant funded by the Korean government (MSIT) (No. 2022R1A2C1008993).

## Acknowledgments

Resources have been provided by Abdul Wali Khan University Mardan (34°12′4.4″N and 72°01′33″E), Pakistan. The present research experiments were mainly carried out at the Department of Botany (PMI Lab, Molecular Biology Lab, Botanical Garden) and the Department of Biotechnology (Plant Biotechnology Lab).

## Conflict of interest

The authors declare that the research was conducted in the absence of any commercial or financial relationships that could be construed as a potential conflict of interest.

## Publisher’s note

All claims expressed in this article are solely those of the authors and do not necessarily represent those of their affiliated organizations, or those of the publisher, the editors and the reviewers. Any product that may be evaluated in this article, or claim that may be made by its manufacturer, is not guaranteed or endorsed by the publisher.
